# Domain-specific physical activity and the risk of colorectal cancer: results from the Melbourne Collaborative Cohort Study

**DOI:** 10.1186/s12885-018-4961-x

**Published:** 2018-11-03

**Authors:** Shahid Mahmood, Dallas R. English, Robert J. MacInnis, Amalia Karahalios, Neville Owen, Roger L. Milne, Graham G. Giles, Brigid M. Lynch

**Affiliations:** 1 Melbourne School of Population and Global Health, University of Melbourne, 207 Bouverie St, Melbourne, VIC 3010 Australia; 2 Cancer Epidemiology and Intelligence Division, Cancer Council Victoria, Melbourne, Australia; 3 Behavioural Epidemiology Laboratory, Baker Heart and Diabetes Institute, Melbourne, Australia; 4 School of Public Health, The University of Queensland, Brisbane, Australia; 5 Department of Medicine, Monash University, Melbourne, Australia; 6 Swinburne University of Technology, Melbourne, Australia

**Keywords:** Survival analysis, Domain-specific physical activity, Exercise, Colon, Hazard ratio

## Abstract

**Background:**

Physical activity reduces the risk of colorectal cancer (CRC), but the relevant evidence derives primarily from self-reported recreational and occupational activity. Less is known about the contribution of other domains of physical activity, such as transport and household. We examined associations between domain-specific physical activities and CRC risk within the Melbourne Collaborative Cohort Study.

**Methods:**

Analyses included 23,586 participants who were free from invasive colorectal cancer and had completed the International Physical Activity Questionnaire-Long Form at follow-up 2 (2003–2007). Cox regression, with age as the time metric, was used to estimate hazard ratios (HRs) and 95% confidence intervals (CIs) for ordinal categories of each physical activity domain.

**Results:**

Adjusted HRs for the highest versus the lowest categories of physical activity were 0.71 (95% CI: 0.51–0.98; *p*_*trend*_ = 0.03) for recreational activity; 0.80 (95% CI: 0.49–1.28; *p*_*trend*_ = 0.38) for occupational activity; 0.90 (95% CI: 0.68–1.19; *p*_*trend*_ = 0.20) for transport activity; and 1.07 (95% CI: 0.82–1.40; *p*_*trend*_ = 0.46) for household activity.

**Conclusions:**

Recreational activity was associated with reduced CRC risk. A non-significant, inverse association was observed for occupational activity, whereas no association was found for transport or household domains.

**Electronic supplementary material:**

The online version of this article (10.1186/s12885-018-4961-x) contains supplementary material, which is available to authorized users.

## Background

Systematic reviews conducted by international and national agencies have concluded that there is convincing evidence that physical activity reduces colon, but not rectal cancer risk [1–3]. Recently, a pooled analysis of 1.44 million adults from across the United States and Europe found that higher leisure-time physical activity was associated with a lower risk of both colon (16% reduction) and rectal (13% reduction) cancers [4].

Physical activity is a modifiable lifestyle behaviour that can take place in different settings (domains). Physical activity can be influenced by personal attributes such as motivation, beliefs, social support from friends and family, as well as the natural and built environment [5]. Correlates of physical activity tend to differ by domains [6, 7]. For older adults living in high income countries (where colorectal cancer [CRC] is highly prevalent), recreational physical activity comprises only a small part of their total physical activity. Previous studies suggest that the activity energy expenditure of older adults is largely determined by physical activity in occupation and household domains [6, 8].

The biological mechanisms underlying the associations between greater physical activity and reduced CRC risk are not clearly understood. Metabolic, inflammatory and hormonal pathways may partially explain how physical activity lowers CRC risk. Low levels of physical activity have been shown to increase blood glucose values and produce insulin resistance and hyperinsulinemia [9]. Insulin may be a key factor in carcinogenesis, due to its mitogenic properties. Insulin has also been described as an essential element for colonic mucosal growth [10, 11]. Increased plasma concentrations of Insulin-like growth factor (IGF) and IGF binding protein-3 provide a favourable environment for cell apoptosis [12, 13]. Regular exercise has a beneficial effect on inflammatory markers such as adipocytokines [14]. Inflammation is widely acknowledged as a risk factor for numerous chronic diseases, including most cancers [15–17].

Most of the evidence for associations with CRC risk comes from studies that have examined physical activity within recreational and occupational domains. The contribution of activity in other domains, such as transport and household has received less attention [18]. Given that physical activity in different domains varies in terms of its frequency, duration and intensity, it is important to elucidate domain-specific associations with CRC risk.

It is also important to understand the role of domain-specific physical activity in relation to CRC risk to help tailor health promotion strategies for intervention, and improve policy guidelines for prevention. In this study, we examine prospective associations between domain-specific physical activity, including activity within the recreation, occupation, transport and household domains, and CRC risk for participants in the Melbourne Collaborative Cohort Study (MCCS).

## Methods

### Study population

**Fig. 1:**
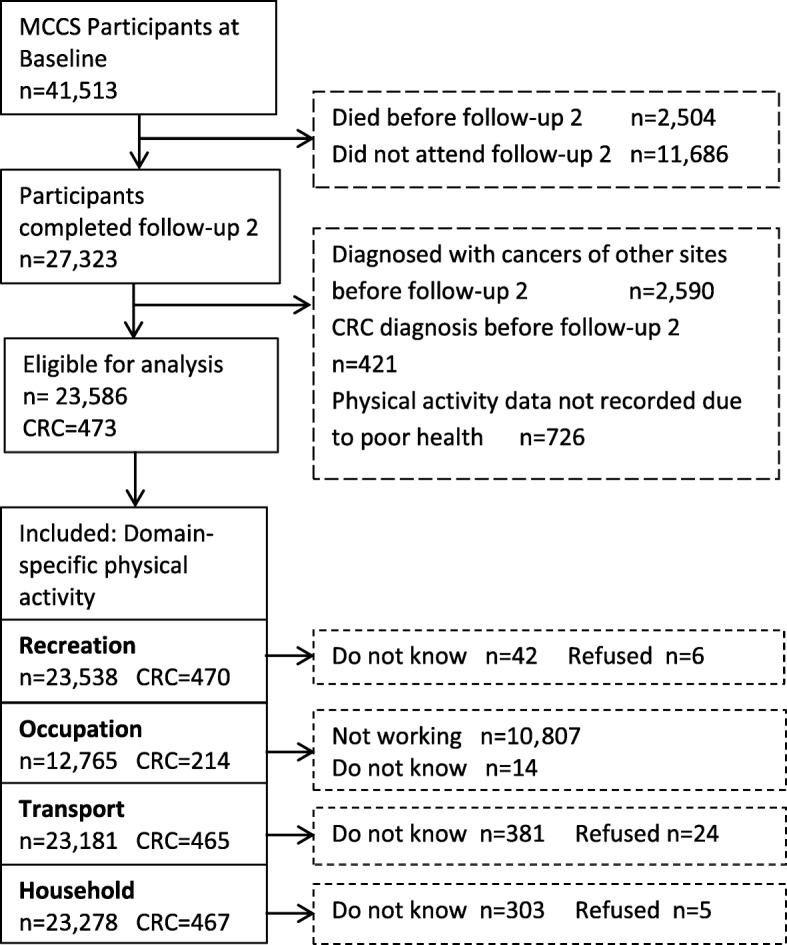
Flow diagram showing the selection process of Melbourne Collaborative Cohort Study participants for the analyses to examine associations of domain-specific physical activity and colorectal cancer risk

The MCCS is a prospective cohort study designed to identify relationships between socio-demographic factors, lifestyle patterns, diet and the risk of developing cancer and other non-communicable diseases. A comprehensive description of the MCCS is available elsewhere [19]. In brief, 17,044 men and 24,469 women aged 27 to 76 years (99.2% were 40 to 69 years) were recruited from the Melbourne metropolitan area between 1990 and 1994 (baseline). Southern European migrants were over-sampled to increase the variability of dietary and other lifestyle factors. Baseline data on physical activity was not domain-specific and did not contain information on duration of physical activity or its intensity. Therefore, we only analysed physical activity data from 27,323 MCCS participants who completed an interviewer-administered questionnaire between 2003 and 2007, which we refer to as follow-up 2. We excluded 3011 participants with prevalent, invasive cancer at follow-up 2, and 726 who did not complete the physical activity section of the interview (see Fig. 1). After these exclusions, 23,586 participants were eligible for analyses related to domain-specific physical activity and CRC risk. For the occupational physical activity domain, we included only the 12,765 participants who were currently working (paid or voluntary). The research protocol was approved by Cancer Council Victoria’s Human Research Ethics Committee [20].

### Ascertainment of exposure status

At follow-up 2, a health and lifestyle questionnaire, including a section on physical activity, was administered in person by trained interviewers. The long-form International Physical Activity Questionnaire (IPAQ) was administered to collect data pertaining to domain-specific physical activity. The IPAQ asks about time spent in recreation, occupation, transport and household domains of physical activity. Within each domain, items relating to the frequency, duration and intensity of physical activity were completed. The reference time frame for these questions was the last 3 months, e.g. “In a typical week during the last three months, how many days per week did you do vigorous physical activities in your garden or yard for maintenance?”, followed by “how much time did you usually spend doing them in a single day?”. Only activities of 10 min’ duration or longer were self-reported.

Metabolic equivalents (METs) within each domain were calculated by multiplying hours per week of physical activity by the intensity level assigned by the IPAQ (long form) guidelines for data processing and analysis [21]. As per the IPAQ guidelines, we truncated time spent walking (transport domain) and in recreational physical activity to 180 min per day for any respondent who reported higher durations, resulting in a maximum of 21 h per week of activity within each of these two domains. For the domains with more than one intensity level assessed (recreation, household), MET hours per week of moderate and vigorous intensity activities were summed to make a single continuous variable. Total MET hours per week in each domain was then categorised into four exposure levels. For occupational physical activity, in addition to the hours per week of paid or voluntary work, participants were also asked to select their usual occupational activity intensity level from an ordinal scale (‘Mainly sitting’, ‘Mainly sitting with occasional walking and moving about to do tasks’, ‘Mainly on feet with some light carrying or lifting’, or ‘Hard physical effort, e.g. scrubbing floors, digging, heavy carrying or lifting’). We used the Compendium of Physical Activities [22], to assign a MET value to the occupational activity intensity level nominated by participants. ‘Mainly sitting’ was assigned a value of 1.5 METs; ‘Mainly sitting with occasional walking and moving about to do tasks’ was assigned 1.87 METs (assuming 75% sitting at 1.5 and 25% on feet at 3.0 METs); ‘Mainly on feet with some light carrying or lifting’ was assigned a MET value of 3.0, and ‘Hard physical effort’ was assigned 6.5 METs. A continuous MET hour per week value for occupational physical activity was derived; this was divided into four categories (quartiles).

### Covariate assessment

Participants completed a structured interview on socio-demographic characteristics, country of birth, education and lifestyle factors including smoking, alcohol, and diet. Residential postcodes were used to assign participants to a quintile of socio-economic status based on the Index of Relative Socio-Economic Advantage and Disadvantage obtained from Australian Bureau of Statistics census-based Socio-Economic Indexes For Areas (SEIFA). Participants attended the study centre to have anthropometric measurements (body mass index [BMI] calculated from body mass measured using Tanita scales to the nearest 0.1 kg, and height measured by stadiometer to the nearest millimetre/half a centimetre; and waist circumference to nearest millimetre) taken by study staff. Dietary data on red meat (beef, lamb, pork), processed meat (bacon, ham, sausages) and total energy intake (including or excluding fibre) were collected using a self-administered 144-item food and beverages frequency questionnaire (FFQ) designed specifically for MCCS. Frequency questions were complemented by the images of food portion sizes. Nutrient intakes per day from FFQ were calculated using nutrient composition data from NUTTAB 2010 [23]. Alcohol intake data were collected by asking beverage-specific questions for frequency and daily consumption. Similarly, question on smoking comprises of never, ever (time quit) and current (number of cigarettes per day) smoking status.

### Follow-up and outcome

Cancer diagnoses were ascertained by record linkage to the population-based Victorian Cancer Registry (VCR) and to the Australian Cancer Database. The International Classification of Diseases for Oncology, 3rd edition, was used to classify all incident colon (C18.0, C18.2-C18.9), rectosigmoid junction cancers (C19.9) and rectal cancers (C20.9). Ascertainment of cancers was complete to 31 January 2016.

### Statistical analyses

We used Cox proportional hazards regression to estimate hazard ratios (HR) and 95% confidence intervals (CI) for CRC risk in relation to recreation, occupation, transport and household domains of physical activity, using age as the underlying time metric. Follow-up (person-time) for this analysis began at the follow-up 2 interview (when the IPAQ was completed) and ended at the date of CRC diagnosis, death, migration from Australia, or 31 January 2016, whichever came first. Participants who had a CRC tumour with a benign, uncertain or in-situ behaviour codes were censored at date of diagnosis.

Proportional hazards assumptions were checked both graphically and statistically for any violation. Global tests, based on Schoenfeld residuals, showed no evidence of major violation for the physical activity exposure variables, or any of the potential confounders.

We initially considered the following variables as covariates to potentially include in multivariable analyses: age (at follow-up 2 interview), sex, country of birth (Australian/New Zealand/UK; Italy/Greece-recruited at baseline as migrant group from Southern Europe), education (primary, some high/technical, completed high school, completed tertiary degree/diploma), socioeconomic position (quintiles), smoking status (never, former, current), total alcohol consumption in grams per day (none, < 10, 10–20, > 20), family history of CRC in first-degree relatives, BMI (kg/m^2^), waist circumference (centimetres); red meat, processed meat and dietary fibre consumption (all as grams per day) and total energy intake (kilojoules per day).

Three sets of multivariable models were fitted to evaluate the associations of each domain of physical activity with CRC risk. The first model included variables identified by using a directed acyclic graph (DAG, see Additional file 1: Figure S1). This first set of models also considered other potential confounders reported by previous studies, including total energy intake, energy-adjusted red meat intake, processed meat and daily dietary fibre consumption. Adding these variables to the models did not materially affect the HRs, and they were not included in our final multivariable models.

Measures of adiposity (BMI or waist circumference) were not included in our primary models because of their potential mediating role (i.e., being in the causal pathway) in the association between physical activity and CRC. However, adiposity might be a confounding factor; the second set of models included waist circumference, which is a stronger predictor of risk of CRC than BMI in this cohort [24]. In the third set of models, missing data were incorporated by multiple imputation using chained equations [25, 26]. To identify auxiliary variables to include in the imputation model, correlations between each of the covariates with domain-specific physical activity were initially explored to identify strong predictors of missingness to be included in imputation model. These predictors, together with the exposure and outcome, were included in the imputation model. The imputation process was repeated 20 times to obtain plausible values for the missing data [25].

For each domain of physical activity, the lowest category was used as the reference. Linear trends across physical activity categories were examined by fitting as a continuous variable the median value for all observations in a given category. Departure from linearity was assessed by comparing the models using domain-specific physical activity as categorical and continuous variable and calculating the *p*-value using likelihood ratio test. Statistical interactions were assessed by introducing interaction terms between domain-specific physical activity and sex, country of birth, alcohol, smoking and waist circumference. Likelihood ratio tests were used to assess these interactions.

Sensitivity analyses were conducted by repeating all analyses excluding cases diagnosed in the first 2 years of follow-up. We used 0.05 as the level of statistical significance and all *P-*values were two-sided. All statistical analyses were performed using Stata version 13.0 (Stata Corporation, College Station, Texas, USA).

## Results

Figure 1 shows the flow diagram illustrating the inclusion and exclusion process of MCCS participants for current analyses. A total of 23,586 participants completed the domain-specific physical activity questions and 473 of those were diagnosed with incident colorectal cancers (336 colon, 25 rectosigmoid and 112 rectal).

Table 1 describes the socio-demographic and lifestyle-related characteristics of study participants. CRC cases had a greater mean age than non-cases (70 versus 66 years), higher waist circumference (92.8 cm versus 90.7 cm) and fewer cases had received a tertiary education (25.2% versus 31.4%).

**Table 1:** Socio-demographic and lifestyle characteristics of participants in the Melbourne Collaborative Cohort Study (MCCS– Follow-up 2)

	All participants	CRC cases	Non-cases
	(*n* = 23,586)	(*n* = 473)	(n = 23,113)
Age at entry (years, Mean ± SD)	65.6 ± 8.7	70.2 ± 8.0	65.5 ± 8.7
Country of birth, n (%)			
Australia/New Zealand/UK	19,376 (82.2)	386 (81.6)	18,990 (82.2)
Greece/Italy	4210 (17.8)	87 (18.4)	4123 (17.8)
Highest education achieved, n (%)			
Primary School	2976 (12.6)	68 (14.4)	2908 (12.6)
Some high/technical school	8970 (38.0)	191 (40.4)	8779 (38.0)
Completed high/technical	4259 (18.1)	95 (20.1)	4164 (18.0)
Tertiary/diploma/degree	7381 (31.3)	119 (25.2)	7262 (31.4)
SEIFA, n (%)			
Ist Quintile- most disadvantaged	3701 (15.7)	76 (16.1)	3625 (15.7)
2nd Quintile	4407 (18.7)	90 (19.0)	4317 (18.7)
3rd Quintile	3703 (15.7)	71 (15.0)	3632 (15.7)
4th Quintile	4640 (19.7)	104 (22.0)	4536 (19.6)
5th Quintile - least disadvantaged	7135 (30.3)	132 (27.9)	7003 (30.3)
Smoking status, n (%)			
Never	14,292 (60.6)	270 (57.1)	14,022 (60.7)
Former	8243 (34.9)	189 (40.0)	8054 (34.8)
Current	1051 (4.5)	14 (3.0)	1037 (4.5)
Current alcohol intake (g/d), n (%)			
None	7768 (32.9)	168 (35.5)	7600 (32.9)
< 10	6184 (26.2)	108 (22.8)	6076 (26.3)
10–20	4283 (18.2)	87 (18.4)	4196 (18.2)
> 20	5351 (22.7)	110 (23.3)	5241 (22.7)
Red Meat intake (g/d), n (%)			
< 30	6097 (25.9)	120 (25.4)	5977 (25.9)
≥ 30-< 45	5901 (25.0)	114 (24.1)	5787 (25.0)
≥ 45-< 75	5778 (24.5)	109 (23.0)	5669 (24.5)
≥ 75	4628 (19.6)	101 (21.4)	4527 (19.6)
Missing	1182 (5.0)	29 (6.1)	1153 (5.0)
Processed Meat intake (g/d), n (%)			
< 4	5788 (24.5)	97 (20.5)	5691 (24.6)
≥ 4-< 8	5461 (23.2)	124 (26.2)	5337 (23.1)
≥ 8-< 20	6090 (25.8)	133 (28.1)	5957 (25.8)
≥ 20	4892 (20.7)	87 (18.4)	4805 (20.8)
Missing	1355 (5.7)	32 (6.8)	1323 (5.7)
Family history of CRC, (%)			
No	20,786 (88.1)	412 (87.1)	20,374 (88.1)
Yes	2359 (10.0)	50 (10.6)	2309 (10.0)
Missing	441 (1.9)	11 (2.3)	430 (1.9)
Waist circumference (cm, Mean ± SD)	90.7 ± 13.0	92.8 ± 12.3	90.7 ± 13.0
Dietary fiber intake (g/d, Mean ± SD)	27.5 ± 9.2	26.5 ± 8.7	27.5 ± 9.2
Total energy intake (KJ/d, Mean ± SD)	8572 ± 2267	8531 ± 2292	8572 ± 2266

Abbreviations: *MET*, Metabolic equivalent; *CRC*, Colorectal Cancer; *SD*, standard deviation; *KJ*, Kilojoules; *m*, meter; *g*, grams; *d*, day; *SEIFA*, Socio-Economic Indexes For Areas. Values are n (%), unless otherwise stated. Percentages are calculated by column

Table 2 shows the estimated hazard ratios (HRs) for the associations between physical activity in recreation, occupation, transport and household domains and risk of CRC.

**Table 2:** Hazard Ratios (95% Confidence Intervals) for the associations between domain-specific physical activity and colorectal cancer risk, Melbourne Collaborative Cohort Study – Follow-up 2 (2003–2007)

Physical activity domains	Cases	Person-years	Model 1^*a*^	Model 2^*b*^	Model 3^*c*^
			HR (95% CI)	HR (95% CI)	HR (95% CI)
Recreation (MET h/wk)					
None	286	132,231	1.00	1.00	1.00
≤ 8	68	38,487	0.85 (0.65–1.12)	0.86 (0.67–1.15)	0.88 (0.67–1.14)
> 8 - ≤24	74	43,206	0.86 (0.66–1.12)	0.84 (0.64–1.10)	0.85 (0.66–1.11)
> 24	42	29,507	0.71 (0.51–0.98)	0.76 (0.54–1.06)	0.71 (0.51–0.98)
P_*trend*_ /P_*departure*_			0.03 / 0.68	0.07/ 0.72	–
Occupation (MET h/wk)					
≤ 16	87	34,181	1.00	1.00	1.00
> 16 - ≤58	52	33,301	0.84 (0.59–1.19)	0.82 (0.57–1.76)	0.83 (0.58–1.18)
> 58 - ≤94	38	36,076	0.79 (0.50–1.25)	0.74 (0.46–1.18)	0.81 (0.52–1.28)
> 94	37	33,660	0.80 (0.49–1.28)	0.78 (0.48–1.26)	0.81 (0.51–1.31)
P_*trend*_ /P_*departure*_			0.38 / 0.68	0.30 / 0.60	–
Transport (MET h/wk)					
≤ 4	117	60,099	1.00	1.00	1.00
> 4 - ≤10	135	59,997	1.15 (0.89–1.48)	1.19 (0.92–1.54)	1.16 (0.91–1.49)
> 10 - ≤20	115	61,682	1.00 (0.77–1.30)	0.98 (0.75–1.29)	1.01 (0.78–1.32)
> 20	98	58,173	0.90 (0.68–1.19)	0.96 (0.73–1.28)	0.90 (0.69–1.19)
P_*trend*_ /P_*departure*_			0.20 / 0.39	0.38 / 0.26	–
Household (MET h/wk)					
≤ 7	107	60,562	1.00	1.00	1.00
> 7 - ≤18	105	63,404	0.96 (0.73–1.26)	0.94 (0.71–1.25)	0.98 (0.74–1.27)
> 18 - ≤36	125	58,438	1.14 (0.87–1.48)	1.15 (0.88–1.50)	1.18 (0.90–1.53)
> 36	130	58,465	1.07 (0.82–1.40)	1.06 (0.81–1.39)	1.11 (0.86–1.45)
P_*trend*_ /P_*departure*_			0.46 / 0.51	0.51 / 0.40	–
Recreation and transport combined (Short form IPAQ)					
≤ 6.5	127	59,938	1.00	1.00	1.00
> 6.5- ≤16.5	123	59,104	1.00 (0.79–1.31)	1.01 (0.78–1.31)	1.00 (0.80–1.25)
> 16.5 - ≤32.5	119	61,982	0.95 (0.73–1.22)	0.94 (0.73–1.23)	0.95 (0.74–1.20)
> 32.5	101	62,406	0.80 (0.61–1.00)	0.83 (0.63–1.10)	0.81 (0.65–1.01)
P_trend_ /P_departure_			0.06 / 0.84	0.13/ 0.91	–

Abbreviations: *MET*, Metabolic equivalent; *h/wk*, hours per week; *SEIFA*, Socio-Economic Indexes for Areas

^a^Model 1: Estimates adjusted for age, sex, country of birth, educational status, SEIFA, smoking status, alcohol intake, and mutually adjusted for physical activity domains

^b^Model 2: Estimates additionally adjusted for waist circumference along with all factors in model 1

^c^Model 3: Estimates with multiple imputation for missing covariates, adjusted for factors in model 1

There was a decrease in CRC risk with increasing recreational physical activity (*P*_trend_ = 0.03) and the highest quartile (> 24 MET hours per week) of recreational physical activity was associated with a 29% lower risk of CRC (HR = 0.71, 95%CI: 0.51–0.98) (Table 2). This HR estimate was slightly attenuated and became statistically non-significant when waist circumference was included in the model 0.76 (95% CI: 0.54–1.06, *P*_trend_ = 0.07).

The HR estimate for physical activity in the occupation domain indicated an inverse association, but this was not statistically significant (HR = 0.80; 95%CI: 0.49–1.28 comparing > 94 with ≤16 MET hours per week), and there was no evidence of a linear trend with increasing activity (*P*_*trend*_ = 0.38). The associated HR estimates for transport (comparing > 20 with ≤4 MET hours per week, HR = 0.90, 95% CI: 0.68–1.19; P_trend_ = 0.20) and household activity (comparing > 36 with ≤7 MET hours per week, HR = 1.07, 95% CI: 0.82–1.40; *P*_trend_ = 0.46) were weaker and not statistically significant (Table 2).

The HRs did not materially differ between the physical activity domains and CRC risk when applying multiple imputation (Table 2) or when excluding the first 2 years of follow-up (results not shown). There were no statistically significant interactions by sex, country of birth, smoking status, alcohol intake or waist circumference (results not shown).

## Discussion

In this Australian cohort of men and women, higher recreational physical activity was associated with a lower risk of CRC. A statistically non-significant risk reduction was noted for occupational activity, whereas no association was found within the transport or household domains of physical activity.

The strengths of our study include its prospective design, small loss to follow-up (only 96 participants left Australia), use of a physical activity measure that assessed frequency, duration and intensity across various domains, and our use of rigorous statistical methods (including complete-case and multiple imputation analyses to handle the missing data).

These findings should be interpreted in the context of a number of limitations. First, approximately one-third of living MCCS participants did not attend follow-up 2. Second, at follow-up 2, a high proportion of the study sample were retirees and so the occupation domain analyses could only include approximately the 50% of participants who were currently working (in either a paid or voluntary capacity). Lastly, physical activity was derived by self-report, which is influenced by social desirability and social approval, which in turn can introduce measurement error, and bias the effect estimates towards the null [27].

The findings for recreational activity in relation to CRC risk are consistent with those reported by previous prospective studies and meta-analyses. In our recent meta-analysis comparing highest versus lowest level of domain-specific physical activity, we observed that recreational physical activity was associated with a 20% (RR = 0.80, 95% CI: 0.71–0.89) and a 13% (RR = 0.87, 95% CI: 0.75–1.01) reduced risk of colon cancer and rectal cancer, respectively [18]. The pooled analysis of 1.44 million adults by Moore et al. [4] reported recreational physical activity to be associated with a decreased risk of colon (90th percentile versus 10th percentile RR = 0.84, 95% CI: 0.77–0.91) and rectal cancer (RR = 0.87, 95% CI: 0.80–0.95) risk.

While there was no statistically-significant association with occupational physical activity, the magnitude of the associations by cancer site were similar to our meta-analysis (RR = 0.74, 95% CI: 0.67–0.82 for colon cancer; RR = 0.88, 95%CI: 0.79–0.98 for rectal cancer) [18], suggesting that increased physical activity in the work place is likely to lower the risk of colorectal cancer and our finding is consistent with existing evidence. There was no significant association between transport-related physical activity and CRC, but pooled estimates from three studies (Hou et al. [28], Takahashi et al. [29] and Simons et al. [30], in our meta-analysis showed a strong association only for colon cancer (RR = 0.66; 95% CI: 0.45–0.98). The null results for household physical activity are consistent with those of our meta-analysis [18], based on a pooled analysis of studies by White et al. [31], Larsson et al. [32] and Friedenreich et al. [14]. The intensity of occupation, transport and household-related activities undertaken by our participants (mean age 66 years) might not be sufficient to impart a cancer prevention benefit. Alternatively, physical activity undertaken within these domains might be more difficult for participants to recall accurately, or subject to unmeasured confounding.

Measurement of physical activity has long been a difficult issue for epidemiological research. Self-report has been the main method employed by researchers to assess physical activity in most large studies. Self-report is subjective by nature, and estimates obtained by this method are also affected by the way in which questions are framed and asked by interviewers. Although the IPAQ has been validated and is widely used in research, there is considerable inter-individual variability in reporting [5], which may be influenced by age and other participant characteristics [7, 33]. This can result in non-differential measurement error and subsequent risk estimation attenuation. Use of objective methods of physical activity assessment (e.g. accelerometers) may reduce systematic biases and measurement error, but due to cost, data processing complexity and participant burden, many epidemiological studies will continue to use self-report instruments.

Researchers have previously applied regression calibration methods, comparing self-report and accelerometer estimates of physical activity, to derive coefficients to ‘correct’ relative risks derived from self-reported data. However, it must be noted in this regard that accelerometers are not gold standard measures. Accelerometers are not able to assess domain-specific activity and may not capture certain activities such as upper body movement or load-bearing, resulting in errors in physical activity measurement [34, 35].

Physical activity is a multifaceted exposure as its pattern varies in different behavioural settings across the life course, and it is influenced by the socio-cultural and built environment. Current public health recommendations emphasise moderate-vigorous physical activity. There is, however, an emerging recognition that light-intensity physical activity contributes considerably to overall daily energy expenditure [6], and thus has potential health benefits such as helping prevent the onset of colorectal cancer. Most of the physical activity undertaken by older men and women comprises of tasks within the transport and household domains [8]. The physical activity of older adults may also be influenced by health status, availability of social support, and access to more conducive environments [36]. It is widely reported that recreational activity decreases with advancing age [37]. Women report significantly more time performing household tasks [6], whereas recreational physical activity only constitutes a relatively small part of total daily activity [8]. While our findings suggested that only recreational physical activity was associated with a lower risk of colorectal cancer, with statistically non-significant associations for occupation and transport physical activity domains; we do not think the findings of our single study should undermine the important role that light-intensity activities play in helping older adults to participate in physical activity and maintain physical function. We also cannot disregard the physical activity measurement issues in our study, especially in the household domain, where activities may be difficult to recall reliably, resulting in random misclassification. This type of misclassification may have weakened the associations of physical activity in transport and household domains with decreased colorectal cancer risk. Device-based measurements can improve the validity of recall and improve accuracy and precision of the estimates [38].

## Conclusions

Recreational physical activity was associated with a reduced risk of CRC. There was a non-statistically significant inverse association for occupational physical activity and no association for transport or household physical activity and CRC risk. Physical activity by older adults within these domains may be of insufficient intensity to confer cancer prevention benefits. These findings corroborate the extant evidence that recreational physical activity is inversely associated with CRC risk. The point estimate we observed for occupational activity was of similar magnitude to that reported previously, but our analysis for this domain lacked statistical power.

Due to the scarcity of research conducted to date, further research focusing on physical activity in transport and household domains is warranted to derive a clearer understanding of whether there are CRC prevention benefits to be gained by increasing activity in these contexts.

## Additional file

Additional file 1: **Figure S1.** Causal diagram showing the potential confounding variables used in the analysis models. (TIF 2735 kb)

## Abbreviations

CI: Confidence interval
CRC: Colorectal cancer
DAG: Directed acyclic graph
FFQ: Food frequency questionnaire
HR: Hazard ratio
IPAQ: International Physical Activity Questionnaire
KJ: Kilo-joules
MCCS: Melbourne Collaborative Cohort Study
METs: Metabolic equivalents
SD: Standard deviation
SEIFA: Socio-economic indexes for areas
UK: United Kingdom
VCR: Victorian Cancer Registry
