# Supplementary figures and images for: Domain-specific physical activity and the risk of colorectal cancer: results from the Melbourne Collaborative Cohort Study
Source: BMC Cancer. 2018 Nov 3;18:1063. doi: 10.1186/s12885-018-4961-x (PMC6215664; doi:10.1186/s12885-018-4961-x)

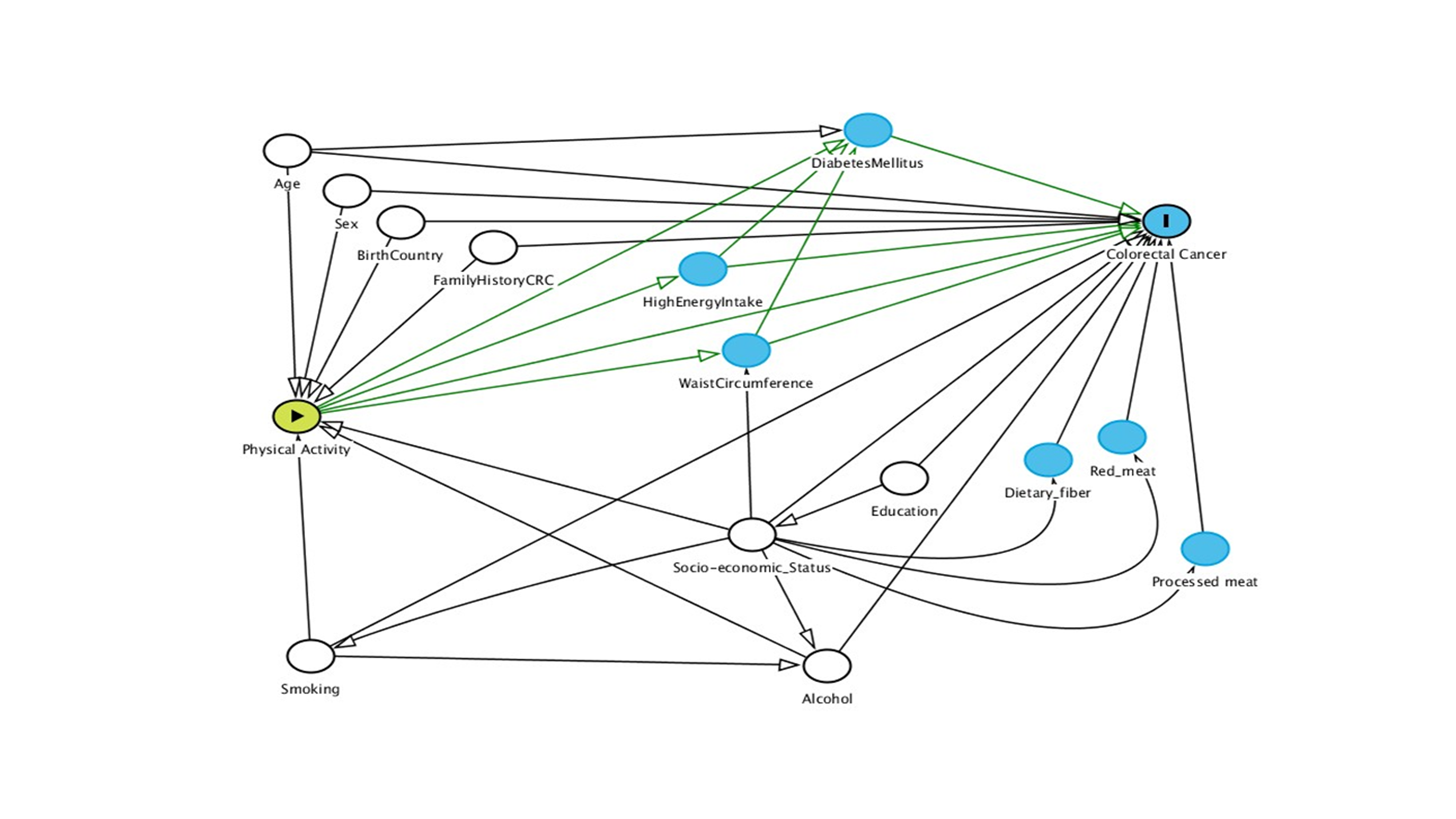

Supplement: Supplementary file 1 — Figure S1. Causal diagram showing the potential confounding variables used in the analysis models. (TIF 2735 kb) [file 12885_2018_4961_MOESM1_ESM.tif]
